# Re-*thinging* Embodied and Enactive Psychiatry: A Material Engagement Approach

**DOI:** 10.1007/s11013-024-09872-6

**Published:** 2024-07-19

**Authors:** Lambros Malafouris, Frank Röhricht

**Affiliations:** 1 Institute of Archaeology, University of Oxford, 36 Beaumont Str, Oxford, OX1 2PG UK; 2 Hertford College, Oxford, OX1 3BW UK; 3 East London NHS Foundation Trust and Queen Mary University of London, London, UK

**Keywords:** Dementia, Schizophrenia, Materiality, Material Engagement Theory, Mental health, Embodiment, Enactivism

## Abstract

Emerging consensus among enactivist philosophers and embodied mind theorists suggests that seeking to understand mental illness we need to look out of our skulls at the ecology of the brain. Still, the complex links between materiality (in broadest sense of material objects, habits, practices and environments) and mental health remain little understood. This paper discusses the benefits of adopting a material engagement approach to embodied and enactive psychiatry. We propose that the material engagement approach can change the geography of the debate over the nature of mental disorders and through that help to develop theoretical and practical insights that could improve management and treatment for various psychiatric conditions. We investigate the potential role of Material Engagement Theory (MET) in psychiatry using examples of aetiologically different mental illnesses (schizophrenia and dementia) in respect of their shared phenomenological manifestations, focusing particularly on issues of memory, self-awareness, embodiment and temporality. The effective study of socio-material relations allows better understanding of the semiotic significance and agency of specific materials, environments and technical mediations. There is unrealised potential here for creating new approaches to treatment that can broaden, challenge or complement existing interventions and practices of care.

## Minding Matter: The Material Engagement Approach to Psychiatry

This paper discusses the benefits of adopting a material engagement approach to embodied and enactive psychiatry. By embodied and enactive psychiatry we essentially refer to the kind of psychiatry which recognizes mental illness (its symptoms/phenomena, causes, and treatment) in the context of what is broadly referred as the extended mind theory (Fuchs & Röhricht, 2017; Fuchs, 2017; Röhricht et al., 2014; Davies, 2016; De Haan, 2020; Hoffman, 2016; Krueger, 2020, 2021; Glackin et al., 2021; Roberts et al., 2019).The extended mind theory comes in different forms (also known as waves) variously based on the principles of parity, complementarity, predictive processing and enactivist dynamics (for a good review see Gallagher, 2017, 2018, 2023). Taken together these principles are also referred to as the 4E (embodied–embedded–extended–enactive) mind (Newen et al., 2018). What unites extended-mind research is a central commitment to a view of mind that is inseparably linked with our bodily and environmentally embedded activity. For embodied-enactive psychiatry mind is made *of* action *for* action. Importantly, mentality is seen as a cognitive ecology that is not limited by the skin (Bateson, 1973; Hutchins, 2010). Given the above construal, mental illness must also be recognised not just as a brain disorder but as an extended and distributed phenomenon: “a disturbance of the patient’s lived body, lived space and relationships with others” and the material world (Fuchs & Röhricht, 2017, p. 135). As such, to understand the *constitution* of mental illness we need to look more widely at the ecology of the brain (Fuchs, 2017; Gallagher, 2017, 2024; Malafouris, 2019b, 2024; Lock, 2018; Palsson, 2016). Adopting such a perspective also brings psychiatry closer to the anthropological approach to mental health that traditionally emphasises the role that social and cultural factors might play in the expression of psychotic symptoms as well as in research, assessment, and treatment of schizophrenia (Csordas, 1990, 2021; Jenkins, 1991, 2015; Kleinman, 1987; Maietta et al., 2020; Myers, 2011; Paul et al., 2020; Whitley, 2014).

The proposed material engagement approach to psychiatry shares these fundamental assumptions for a dynamic topology of the ‘mental’ and a radical continuity between perception, cognition and action. What Material Engagement Theory (henceforth MET) adds to the mix is a set of hypotheses, concepts, descriptions and methods that allow new ways of thinking about the continuity and co-constitution of mind and matter. In addition, rooted in cognitive archaeology, MET brings a distinctive sustained anthropological attention and comparative focus on the material conditions of human cognitive becoming (development and evolution) (Malafouris, 2013, 2019a, 2020; Malafouris & Renfrew, 2010).

In particular, MET can be described by means of three major and complementary working hypotheses about cognition, signification, and agency (for detailed discussion see Malafouris, 2013, 2019b, 2020): (1) *the hypothesis of the extended mind* argues that human mental life is constrained and constituted by our evolved bodily and neurophysiological makeup as well as from our changing material environments; (2) *the hypothesis of enactive signification* essentially propose that meaning-making is not the *pre-formed* product of symbolic representation but of a *performed* enactive conflation and co-habitation of the signifier and the signified. This represents essentially a semiotic of matter that moves beyond representation and language. Enactive signification is an expressive and creative process that allows meaning making in a lived primary sense. Last, (3) *the hypothesis of material agency* basically states that agency is not a human property but the emergent product of situated activity. What an entity is in itself does not really matter; what does matter is what it becomes and where it stands inside a community of action and material engagement (Malafouris, 2008a, p. 34; Knappett & Malafouris, 2008).

Those three hypotheses, that constitute the theoretical foundation of MET, form a unity and should not be separated. Each of them informs, complements and supports the other two. Taken together they offer the conceptual and empirical means for re-describing thinking as *thinging*. The main argument here is that people think and feel *with* and *through* things; not just *about* them (Malafouris 2019b, 2020). Moreover, the thinking *with* and *through* precedes (ontogenetically and ontologically) the thinking *about*. The term *thinging*, is used to denote this distinctive ecology of the human *hylonoetic* field (from Greek *hylē* for matter and *noêsis* for intelligence). No other lifeworld can be argued to be substantially constituted on the basis of its changing relationship with the variety of material objects and technologies that it makes and uses (Malafouris, 2024; Ihde & Malafouris, 2019). Humans are creatures of this peculiar sort: we create things that very often alter the ecology of our minds, re-configure the boundaries of our bodies and the ways we make sense of the world. This is also known as *metaplasticity*: human becoming (nature and culture) is inextricably enfolded with the plasticity the changing socio-material environments (Malafouris, 2008b, 2010, 2013, 2015; Aston, 2019; Roberts, 2016). The driving force here being the plasticity of the brain linked to the affordances of our body and the action possibilities offered by the things we make and use in different contexts.

MET provides a cross-disciplinary methodological tool designed to facilitate a better understanding of how specific human–material entanglements emerge and are re-enacted or transformed over time through practice. This opens up new possibilities for exploring the cognitive life of things (Malafouris & Renfrew, 2010; Sutton, 2008) and understanding the efficacy of materiality and material culture in human mental functioning and constitution. As a consequence, the range of phenomena and processes that psychiatry can and should take into consideration in order to understand a person’s mental health is radically expanded. This expansion can help psychiatry make better sense of mental habits, practices and classifications as well as of the possible mechanisms that form and sustain them. This broadening of perspectives and analytical units builds on earlier work in the anthropology of distributed cognition (Goodwin, 2000; Hutchins, 2010) and is very much in line with both old and new enactive–ecological trends in philosophy and embodied cognitive science (Clark, 1998; Gallagher, 2017; Newen et al., 2018). However, a key methodological difference remains: Despite having recognised the importance of the wider extra-neural environment, these philosophical frameworks remain preoccupied with important but rather abstract analytical issues and debates, thus, treating material culture as secondary, or simply regard it in very general terms (Malafouris, 2018). As a result, the agency of things (intra-action potential) remains an abstraction with little or no real impact on how we understand and treat specific mental disorders. Similarly, although the peculiarity of the human lived body as experienced both from the ‘inside’ and from the ‘outside’, as well as the kinesthetic and social dimensions of bodily movement and intersubjectivity are well studied, the ‘situatedness’ of that body inside its relevant material environment remains little understood (Malafouris, 2024).

The main differentiating feature of the proposed material engagement approach lies precisely in its conviction that attention to material things (we use the term things in the broadest sense of objects, materials and environments) and to the modes of our everyday engagement with things (our habits and material practices) is critical if we are to tackle effectively the contemporary challenges of mental health (i.e. primary and secondary prevention, promotion of well-being, better understanding and management of mental illness by using creative interventions that extend beyond clinical treatment). Indeed, if we accept the full claim of active externalism, namely, that parts of the world *are* potentially parts of the mind (rather than merely *causally* connected to it) then, (a) examining exactly *when* and *which* parts are constitutive of *what* mental processes, and (b) translating the insights that may come out of this new understanding in psychiatric terms, across the mental health continuum, seems to be the way forward for making progress with those issues. We should also explain that the meaning of ‘situatedness’ in the context of MET is both materialist, pragmatist and, above all, process-based (emphasising modes of *becoming* rather than *being*). When we speak of cognition as extended and situated we refer to an active diachronic participation and co-constitution of ‘internal’ and ‘external’ parts that form, in their continuity, the major characteristic of human self-*becoming*. This applies both from an evolutionary and an ontogenetic (developmental) perspective. It should be also emphasised in this context, that the meaning of the term ‘interaction’ when we speak of interactions between mind and matter or persons and things can have different meanings (some more radical than others). In particular, the interactivity of material relations (where mind, society and matter exchange properties) can be more accurately described through notions of ‘trans-action’ (as defined from the pragmatist point of view by Dewey & Bentley 1949), or ‘intra-action’ (as defined from the agential realist perspective of Barad, 2003). The key difference expressed here, which applies both from an evolutionary and a developmental point of view, is that the entities to be related (mind and matter or persons and things) and the boundaries that will be used to distinguish and define those entities can only emerge *after* the interaction (that is, they do not predate their entanglement). This primacy of active engagement over passive objects also indicates why material environments are always relational. They are relational because they are not made of fixed properties; instead, they are made of action possibilities and situational affordances. This relationality gives meaning to the condition of human ‘situatedness’. A material environment is not what surrounds the person’s thinking—whatever happens to exist ‘out there’ rather than ‘in here’, passively enveloping or actively mediating. Rather, it denotes the semiotic *hylonoetic* field where brains, bodies and things are intra-actively produced.

Being fundamentally a temporal and situated process*,* self-becoming is locationally uncommitted (Malafouris, 2024). Obviously such a decentralised conceptualisation changes the geography of the debate over the nature of mind and selfhood. Our boundaries are shifting (stabilized or destabilized) in response to our actions and the affordances of specific material environments. Adopting such a decentralised material engagement perspective can help psychiatry integrate the reflective and pre-reflective dimensions of self, and understand better their evolution, their different cultural manifestations as well as the impact that different material ecologies have on human mental life. The basic idea is that altering the person's environment can result in changes (positive or negative) in embodied self-awareness and the modes of *thinging*.

This shift retains a person-centred approach to care (the patient’s ability to regain their agency and control of their bodies to undergo their daily activities remains the main objective). However, it argues for a de-centred model of understanding mental health problems and their treatment (recognising that the patient’s agency and bodily control that form the foundation of mental health and well-being are not properties of the individual) (cf. Gibson et al., 2021). We should be reminded here of Gregory Bateson’s famous moto, published already in 1971 in his paper on ‘The Cybernetics of Self’ that “*the mental characteristics of the system are immanent, not in some part, but in the system as a whole*” (italics in the original). Our attempt at decentralising subjectivity is part of our widening strategy for accommodating the agency of the material environment in psychiatric disorders and closing the gap between the cognitivist model and our everyday experience of selfhood. More factors than those we traditionally identify as constituting the ‘disease’ can be causing each of the separate symptoms. The value of that for psychiatry is precisely that it allows us to integrate the manifold and often heterogeneous symptoms of psychotic experiences avoiding the need to localise mental illness inside the patient’s head (Fuchs & Röhricht, 2017, p. 135).

Bringing anthropological and archaeological sophistication on the study of material culture to bear on the proposed re-thinging of ‘psychiatric’ approach to mental illness can be mutually enriching in helping us understand the profoundly embodied and situated character of human self-experience. “The human subject”, as Danny Miller well points out, “cannot be considered outside of the material world within which and through it is constructed” (Miller, 1987, pp. 86, 214). Things are widely recognised as active, creating dependencies and opportunities for action (Knappett & Malafouris, 2008; Gell, 1998; Hodder, 2011; Latour, 1991). They both mediate social interaction and guide human action in specific directions as when a speed bump forces drivers to slow down (Latour, 1992, 2005). Defining the social interactions between humans are no longer privileged over transactions between humans and things. In fact, it is impossible to conceive of material culture outside sociality and of sociality in the absence of material mediation (Latour, 1994, p. 806, 1999, 2005). Understanding the cognitive (Malafouris & Renfrew, 2010; Knappett, 2005), social (Appadurai, 1986; Miller, 2010; Gosden, 2008; Latour, 1999, 2005), and evocative life of things (Turkle, 2011) has been a central theme of research within anthropology, archaeology and sociology. Material culture has been shown also to have strong affective significance, offering comfort and emotional support (Miller, 2008). There has been also fertile discussion and debate relevant to the issue of material and non-human agency: in what sense, if any, can it be argued that things have agency? (Bennett, 2010; Gell, 1998; Knappett & Malafouris, 2008; Ingold, 2008). Is it that things have agency in a ‘secondary’ derivative sense (Gell, 1998), where they only seem as agents because they extend the agency of the humans that create or use them, or is that they are literally agents in a ‘primary’ sense, identified as animate forces of their own (Alberti et al., 2011; Bennett, 2010; Kohn, 2015)? MET occupies a middle position in this debate of the new materialist (Bennett, 2010; Coole & Frost, 2008) and posthumanist vision (Latour, 1992; Barad, 2003; Pickering, 1995) of matter as agentive and vibrant. As we discussed, from a material engagement perspective, agency is not the kind of phenomenon that can be described, let alone accounted for, by looking either at the human or the non-human alone. Instead, agency can only be the emergent relational product of their entanglement. Recognising that things matter and must be taken seriously (Miller, 1998), the question that follows is what are the implications for understanding basic self-disorders like schizophrenia and dementia? Adopting the material engagement approach, our basic aim is to provide a starting point that allows us to *join forces with matter*, highlighting the intimate connections between humans and things. We argue that psychiatry should take advantage of those intimate relationships and try to understand the full variation of the interaction between cognition and material culture by avoiding setting a priori boundaries and by being responsive to the nature of the phenomena under study.

Responses to different forms of materiality can guide or inform the design of settings for promoting patient recovery and treatment (Fuchs & Röhricht, 2017; Moser, 2011; Stephens et al., 2013; Buse & Twigg, 2018; Maller, 2015; Cleeve et al., 2020). Consequently, this paper investigates some key aspects of our engagement with the world focusing on the examples of dementia and schizophrenia which constitute paradigm cases for exploring the material bases of psychopathology.

## Why Schizophrenia and Dementia?

In order to explore the impact of MET for psychiatry, we have chosen two disorders, schizophrenia and dementia, given their phenomenological common ground of severely altered self-awareness, and their distinct aetiological nature across the spectrum of biological and psychosocial causalities. Schizophrenia and dementia both result in profound consequences on patient’s abilities to meaningfully and effectively relate to the world (material engagement problems). At the same time there is very little evidence of shared pathophysiologic mechanisms between them (Cooper & Ovsiew, 2013), allowing us to explore impacts of the MET in two aetiologicaly distinct disorders with a degree of shared psychopathology.

Despite their diverse neuropathology and symptomatology, both schizophrenia and dementia relate to, and can be identified as severe self-disturbances. The spectrum of schizophrenic conditions is traditionally understood and unified, especially from the point of view of phenomenological psychiatry, as a self-disturbance associated with pre-reflective bodily or minimal self (Gallagher, 2000, 2024; Gallese & Sinigaglia, 2010; Sass & Parnas, 2003; Fuchs & Schlimme, 2009, Fuchs & Röhricht, 2017). In the case of dementia the underlying pathology is often described as a diminishment of or loss of self (Kontos, 2004; Kontos & Martin, 2013; Millett, 2011; Mograbi et al., 2021; Moser, 2011) and is seen as the result of severe memory disfunctions. It can be argued, however, that loss of memory does not necessarily equate to a global loss or decay in the processing of self-related information and subjective self-continuity across the dementia syndromes (Strikwerda-Brown et al., 2019).^1^

Schizophrenia and dementia are considered two of the leading causes of disability worldwide (with no effective drug or other treatment yet available in most cases) forming a major challenge to modern society. We believe that, fostering deeper anthropological and phenomenological understandings about what it is like to experience and to live with dementia and schizophrenia, as well as about the possible variation (and local specificity) in the symptoms, course, and outcomes for people diagnosed with both disorders (Myers, 2011) is the way forward in order to maximise opportunities for the search for new and effective management approaches. We argue that a material engagement approach, focusing on the diverse ways human self-experiences are entangled in, and co-constituted through, relations with other humans and things, can provide a refined ecological understanding of how the situated minimal and narrative self are affecting and are affected with the onset and progression of both diseases. There is unrealised potential here for creating new approaches to care and intervention. These approaches can complement, broaden, or even challenge existing practices leading to the development of new interventions enabling people to improve their sense of agency and manage their impairment.

In the following sections we shall be taking particularly into account disturbances of memory and self-awareness relevant to (a) the self-transformation that is often described as a *diminishment of* or *loss of* self in the case of dementia (Kontos & Martin, 2013; Mograbi et al., 2021; Moser, 2011), (b) disturbances in embodiment, often referred to as a form of disembodiment in the literature (Fuchs & Röhricht, 2017), and temporality (e.g. disruption of the link between time as pre-reflectively lived vs. consciously experienced) in the case of schizophrenia (Fuchs, 2013; Vogel et al., 2020). Attention is drawn, for example, to how guided and creative engagement with selected materials and personal objects can reinforce the sense of agency and offer a connective link between lived time and biographical time that supports self-awareness and the continuity of pre-reflective bodily self and narrative self. The ability of creative material engagement to facilitate self-bounding and temporal anchoring (in addition to non-verbal communication) could prove beneficial for patients with self-disturbances. Bringing things to mind, anthropology and anthropological archaeology have been making extensive use of the “biographical object” approach (in the sense of Hoskins, 1998), where the thingness of objects provide anchors for enactive imagination, introspection, and reflection allowing people to construct self-narratives and meaning in ways that would have been impossible without it.

## Biographical Objects: Mending Demented Life Histories

Notions of object biographies and life histories are well established in anthropology and archaeology. They have been originally introduced to denote the widely shared understanding that things, like humans, undergo a social life (Appadurai, 1986) during which they accumulate histories and cultural biographies (Kopytoff, 1986) that can be recalled and expressed as narratives or object life stories (Gosden & Marshall, 1999; Hoskins, 1998, 2006). *Remembering for us, through and with us*, familiar from the phenomenology of place and memory (Casey, 2000) is not unlike the kind of transactional remembering we observe between humans (Harris et al., 2014). In particular, through their use and intimate associations with humans (mediating basic technique and habits) things provide both material scaffolds for re-enacting (thus remembering) skilled actions, and material anchors for autobiographical events to which they have been linked in the course of their life history. Importantly, objects although tangible and durable are also process-like and dynamical entities mutually constituted with the people who own them and use them and whose lives and memories they actively affect and mediate (Malafouris, 2013, 2015; Gosden & Malafouris, 2015; Miller, 2005; Turkle, 2011). One of the most important lessons anthropology in the last three decades teaches us regarding the cognitive life of things (Malafouris & Renfrew, 2010; Sutton, 2008) is that “as people and objects gather time, movement and change, they are constantly transformed, and these transformations of person and object are tied up with each other” (Gosden & Marshall, 1999, p. 169). Or as the anthropologist Janet Hoskins observes (1998), the biographies of objects and people’s life histories are often intertwined and, thus, biographical objects are “endowed with the personal characteristics of their owners” (1998, p. 7). Hoskins’ extensive ethnography among the Kodi of the Eastern Indonesian island of Sumba, offers also some interesting methodological observations. As she admits, quite to her surprise, she soon realised that she “could not collect the histories of objects and the life histories of persons separately. People and the things they valued were so complexly intertwined they could not be disentangled”. Still, this initial frustration proved to be an advantage in disguise. As she points out: “I obtained more introspective, intimate, and ‘personal’ accounts of many peoples' lives when I asked them about objects, and traced the path of many objects in interviews supposedly focused on persons”. Traditional ‘person-centered’ ethnography had “to be rethought as one that uses objects as metaphors to elicit an indirect account of personal experience” (1998, p. 2).

With MET we could move a step further and ask a more challenging question about the role of things in human self-narrative and memory. That is, we can ask how *objects tell the stories of people’s lives* rather than people telling the stories of objects. This also indicates why it is important to be attentive of both meanings that the term 'object biographies’ may have: on the one hand, as biographies created by people about objects, and, on the other hand, as biographies of people created *with* and *through* objects. We embrace the possibility of a transactional mnemonic entanglement between people and things (Malafouris, 2019b; Malafouris & Koukouti, 2018; Prezioso & Alessandroni, 2022). Things can provide powerful media for recollection, self-expression and self-identification. In that sense, things provide a durable, and often portable network of material signification which can be harnessed to facilitate sense making and compensate for memory loss when biological memory is damaged (e.g. Hamill et al., 2012; Malafouris, 2019b).

A simple, but indicative example, of the importance of the broader ecology outlined above, is what we see during periods of major transition, for instance, during the transition from home to a long-term care facility, often marked by anxiety, confusion and distress caused, among other things, by the changing cognitive ecology and the stripping away of personal possessions that help to constitute one's personal memories and self-identity (Cram & Paton, 1993; Araujo et al., 2020). The philosopher Daniel Dennett, in his book *Kinds of Minds* (1996, pp. 138–139), describes this cognitive impairment when elderly people are in hospital, bereft of the usual contextual cues and affordances of their familiar home environments. To lose these objects, often abruptly and unwillingly, owing to changes in residential circumstances, can be a major cause of loss for elders (Phenice & Griffore, 2013; Wapner et al., 1990). This powerful affective linkage of material possessions to one's identity, the close association between memorabilia and mood, explains the agency of the former on how elderly individuals re-enact their sense of self-continuity and their ability for mental time travel. We argue that those personal objects could be introduced in a systematic manner in the therapeutic process. Carefully chosen evocative objects (Hamill et al., 2012; Phenice & Griffore, 2013; Sherman & Dacher, 2005; Stephens et al., 2013; Treadaway, 2022; Turkle, 2011) can provide a window into the patient’s lives and personal experiences. Objects can potentially be harnessed to support and promote patients’ agency, autonomy and ability in social interaction and communication. Understanding better the importance of object ‘memories’ can allow us to develop strategies of care for the things that can actively enhance or even create a sense of continuity, stability, and comfort (e.g. during those transitional stages when one moves from home to a long-term care facility). Wood (2009) through an interpretive phenomenological exploration of children’s material culture has illustrated the ways by which childhood experiences can be ‘saved’ within everyday objects which can be used later in life to re-enact personal meaning, and potentially regain a lost sense of self. She argues that childhood objects (defined as specific things—not necessarily toys—that an individual has kept or continues to use roughly up to age 12), more than other personal possessions, “act as temporal guideposts to personal development, meaning and identity” (p. 152). Focusing on the lived experience of object transactions, Wood was able to describe the mnemonic agency over multiple generations using examples of simple everyday objects saved from childhood. Important to note here is that studies on bodily and object ownership have pointed out that personal objects enjoy a special processing status (e.g. personal objects trigger a strong memory trace) and are often treated as psychological extensions of self (Cunningham et al., 2008; Van den Bos et al., 2010; De Preester & Tsakiris, 2009).

Another promising avenue of research could be to investigate the cognitive mechanisms (neural and extra-neural) activated when people think *with* and *through* things and compare them with the cognitive mechanisms activated when they think *about* things. Understanding those processes is important, because it may allow us to differentiate between forms or stages of mental illness that seem to affect the latter capacities for thinking *about* the world (in the sense of abstract recollection, self-narrative, communication and language use) while leaving the former enactive capacities largely intact. This could help the development of therapeutic interventions where the latter unaffected enactive capacities for thinking *with* and *through* could be re-used to compensate for the lost ones. A good example of the efficacy and cost-effectiveness of such simple interventions can be seen is the form of memory books, photo albums and so-called memory boxes which provide powerful external memory aids and communicative means precisely because they do not require conscious cognitive effort in order to trigger retrieval of related semantic information from memory storage, effecting positive changes in the conversational behaviours of people with a wide range of severity of cognitive impairment (Bourgeois et al., 2003; Caddell & Clare, 2011; Bae & Kim, 2018; Hamill et al., 2012).

The crucial questions here concerns the extent to which memory loss in dementia and other neurodegenerative diseases might be partially compensated, or scaffolded by the use of selected biographically salient objects and material scaffolds for self-narrative and autobiographical memory (see also Heersmink, 2022a, 2022b). One could look specifically at self-narratives evoked and constructed through participatory sense making with personal artefacts and objects.

## Re-embodying the Self: Objects as Tools for Self-grounding and Temporal Anchoring

According to phenomenological accounts, disturbances of the basic sense of self (ipseity, mineness) are considered as the fundamental disorder in schizophrenia, also described as a disembodiment where the basic ability for a pre-reflective engagement with the world is disrupted (Fuchs & Röhricht, 2017). As a consequence of this weakening of pre-reflective bodily enskilment the relationship of self and world is severed. Basic forms of intra-action and participatory sense-making are now in need of conscious monitoring and deliberate or reflective reconstruction. As summarised by Fuchs (2013), in all its phases, schizophrenia consists in a disturbance of self-constitution “accompanied by profound desynchronisations of intersubjective temporality” which culminate in thought withdrawal or insertion, hallucinations and delusions. This leads to “growing perplexity and hyperreflexive ruminations” (Fuchs & Röhricht, 2017). There is a temporal fragmentation of basic self-experience that “needs to be compensated for” usually by means of “rational reconstruction” and explicit story telling. The purpose of those reflective self-narratives is to re-establish the lost connectivity with the temporal structure of consciousness.

Disturbances of embodiment and participatory sense-making result in various basic dis-continuities between cognition, perception and action. It has been suggested that these disorders can be impacted upon “if one succeeds in changing one’s way of interacting with the world” (De Haan, 2020, p. 11). Gallagher (2018), adopting a similar enactive–ecological perspective, proposes an affordance-based approach to therapy that places special focus on the changing physical and socio-cultural environments. He argues that a variety of neurological and psychiatric disorders can be understood and potentially treated in terms of changes in a subject's ‘affordance space’—which is the lived space defined relative to the capacities (sensorimotor, social, cultural, cognitive) of a specific agent. Recent attempts to provide scaffolding experiential frameworks for patients to explore externalised manifestations of hallucinatory sensations and to practice social interaction in virtual reality paradigms offer concrete steps in that direction (e.g. du Sert et al., 2018); our proposal, however, takes this thinking a step further. Creative re-*thinging* may help patients improve their lost sense of self-coherence which we regard to be a generative disturbance in schizophrenia. In particular, the process of *thinging* (thinking and feeling *with* and *through* things) can support the patient’s disembodied and disconnected patterns of sense-making to achieve a more stable pattern of enactive signification (meaning-making through things) and material imagination. In other words, *thinging* supports the affective and pre-reflective aspects of selfhood, strengthening the overall sense of self-bounding and grounded relatedness with the world. One such example is the creative material engagement with drawings and (clay) sculpturing of dissociated and fragmented body images. Joraschky et al. (1998) demonstrated the utility of human figure sculpturing processes, allowing patients to engage with concrete manifestations of their body image disturbances through tactile and visual amendments of the figures. Röhricht and Priebe (1996) developed and evaluated a group therapy manual for chronic schizophrenia; the intervention include material engagement tasks such as exploring mismatches between perceived and actual physical and kinesthetic space taken up by the bodily dimensions by laying ropes around the body on the floor and vary space requirements. A sense of a weakened and invaded body-self boundaries, associated with self-disorder symptoms of disturbed agency or identity, can be addressed in body-oriented psychological therapy (Röhricht et al., 2009, 2011) with the construction of safe spaces in the therapy room (using a variety of props such as sticks, robes, basic construction materials) as a prerequisite for role play scenarios (e.g. “visiting my neighbour”, “opening doors”, “communicating with others from within the safe and guarded space”). From an embodied mind theory and neuroscientific perspective Sevos et al. (2013) cited studies that indicate how motor (action) information is incorporated in perception (of objects), suggesting that the affordance presented by the object immediately translates into action mapping. Their experiments showed that object-based affordance effects were not found in the schizophrenic group, concluding “it would seem that the automatic binding between perception and action does not take place” (p. 6). It has been argued that dis-automatisation phenomena may result from patients’ overemphasis on self-observation (hyper-reflexivity). Therapeutically patients therefore require assistance in grounding their attention towards action binding perception of their bodily awareness.

Schizophrenia is also often characterised by a fragmentation and weakening or freezing of lived time (in Bergson’s sense of *durée*). As a result of that, these patients are losing the basic affective attunement to the world, and become instead preoccupied or fixed with rational and intellectual aspects; what Fuchs describes as “an arrest of existential temporality” (2013). *Thinging*, in the sense of thinking *about* things, also relates to the reflective or narrative aspects of self and facilitates intersubjective synchronisation. Temporal fragmentation is an area where better understanding of the cognitive life of things may prove especially effective in re-calibrating the protentional–retentional coherence of consciousness (Husserl, 1966) in self-disturbances. In particular, personal and biographical objects can fill in the temporal micro-gaps (i.e. the fragmentation of the temporal sequence of events) which could be understood as being responsible for the experienced thought blockages, interferences, and insertions. It can be hypothesised that things’ ability to intrude into these temporal gaps protects the patient from the usual insertion of disturbing thoughts (leading to experiences of external control and passivity phenomena) which now have no space left to invade or reason to exist. We are so well habituated into allowing things to steer our movements that no further agency (besides the affordances of things) is needed. It is in this sense that personal objects can provide a protective shield against the depersonalisation caused by temporal fragmentation. Embodying multiple temporalities they can provide concrete anchors for grounding time-consciousness and serve as media for building connections across the scales of time, helping patients develop a sense of ‘contemporaneity’ and ‘synchronicity’ through the tactile sensing and sharing of time. Embodied *thinging* can compensate for the lack of the ability to synchronise, thus, help to avoid alienation and emotional and social withdrawal in schizophrenia.

Another example for interventions to address reality and temporal binding distortions relates to clinical observations of one of the many behavioural, i.e. hoarding phenomena that occur in schizophrenia. Schou et al. (2020) examined the hoarding among 13 patients diagnosed with a schizophreniform disorder, which started early in their life and appeared to be related to a diminished sense of basic self and transitivistic experiences (cited by several patients as motivations for collecting objects). They reported that patients described a lack of inner core and how their excessively collected items (belongings) supported them in “feeling present in the common, material world, and in keeping with the continuity of their existence in time. Hoarding may be envisaged as a kind of compensation for fundamental disturbances of subjectivity” (p. 115). Chosen objects can therefore be utilised therapeutically as anchor points for patients’ sense of existence, visual timeline constructions with objects provide experiential frameworks through with the therapist can help patients consolidating their basic self.

## Implications for Psychotherapeutic Practice: The Example of Clay Work

Clay is a morphogenetic material with long and diachronic presence in human becoming (human the species and human the person). We no longer live in worlds made of clay. Still, the craft of ceramics is widely practiced and the material of clay has been used extensively in the context of creative art therapies. It has been shown to help patients diagnosed with mental illness to connect and communicate (often in novel ways) with their selves, their bodies, their friends and therapists, as well as their surrounding environment. In particular, research demonstrated that clay art therapy can aid emotion regulation (e.g. Nan et al., 2023), facilitate self-expression (Abramowitz, 2013), holistic self-experiences (Sholt & Gavron, 2006), and healing from trauma (Elbrecht & Antcliff, 2014). In addition, it has been shown that clay art therapy can help patients to understand their psychotic experiences (Hanevik et al. 2013), reducing negative mood and improving mental well-being (Argyle & Winship, 2018; de Morais et al., 2014; Fancourt & Finn, 2019; Kimport & Hartzell, 2015; Nazari et al., 2018; Timmons & MacDonald, 2008; Wong & Au, 2019; O’Brien, 2024) as well as readiness to change (Leone et al., 2018). So how exactly does it do it? How are we to understand the potential therapeutic qualities of clay? We summarise some characteristic features and affordances of this material that could help advance our discussion of the links between psychiatry and material engagement.

The plasticity and tactility of clay should be mentioned first. Clay can be handled (creatively or not). It can be directed or followed. It can be deformed and reformed. This facilitates non-verbal expression and enactive discovery within a safe space which, in turn, may stimulate dialogue between maker, material and therapist. Another important feature of clay is that it invites attentiveness (Malafouris & Koukouti, 2022), especially attentive listening which can also prove beneficial for the therapist-patient relationship. Moreover, through the handling of clay the patient’s disorganized thoughts and actions as well as uncontrolled movements can be given an expressive direction and a sense of integration, coherence, purpose and containment (Henley, 2002; Röhricht, 2015; Meighan, 2021; Sholt & Gavron, 2006).

Different possibilities and limits of self-experience can be tested within the lived space of this form generating material. New meanings and affordances can be enacted. The creative handling of clay can change both the body schema (extending it) and the body image (allowing enactive material imagination through clay). Overall, clay is an exploratory and playful material (March, 2019, 2024a, b). It resists but it is also accommodating. Clay allows sufficient amount of control while offering endless possibilities for transformation. Moreover, as long as clay remains plastic, whatever happens can be reversed or turned into something else. This feeling of controlled malleability can for example prove beneficial in cases of melancholic depression where, as we discussed before, “time becomes explicit to such an extent that it turns into a constant burden of guilt and omission” (Fuchs, 2010). Clay affords openness, novelty, and surprise, which provide a way to enact and reconceptualise past, present and future as possibilities for action. It also opens up new possibilities for meaning-making (via enactive signification) (Malafouris, 2020; Malafouris & Koukouti, 2022; Malafouris et al., 2023; Koukouti & Malafouris, 2020).

It is important to underline that engagement with different materials brings forth a variety of inter/intra-active possibilities and therapeutic affordances (some of which are better known than others) (Rankanen et al., 2022; Kimmel & Groth, 2024). Consider this characteristic example: it has been reported that schizophrenic patients tend to avoid making spontaneous use of sculpting materials (such as clay or plasticine) (Foster, 1997). They may use clay to make copies of objects but they tend to avoid creating imagery of three dimensional ‘bodies’. This is reflected also in their drawings, which show little differentiation between foreground and background. The interesting difference here is the following: in drawing, forms can be blurred or made to disappear by painting one line on top of another. By contrast, the three-dimensionality of clay means that you start with a material that can be transformed but cannot be made to disappear. Foster describes that as “fear of three-dimensionality” (1997, p. 55). As she describes: “[b]oth the response by my psychotic patients to the initial wet stickiness and to the subsequent drying up, hardening and cracking states of clay on the skin, was usually one of distress and necessitated urgent removal of clay from the skin….It is as if the substance is experienced as continuing to interact with the psychotic patient’s body, even when he or she has discontinued handling it” (Foster, 1997, p. 54). She proposes that “the ‘life-likeness’ and affective aspects of object relations with three-dimensional bodies are of major importance for an understanding of many psychotic patients’ avoidance of working with three-dimensional substances” (Foster, 1997, p. 55).

Important to point out in this context is a common problematic assumption, shared by many art therapists, that the objects made are symbolic containers or projections of the schizophrenic patient’s fragmented self. The patient, on this construal, is often seen as projecting or externalising self-parts into the made object or use it also as a symbolic medium to relate to the therapist. We are not denying that a participatory (and potentially therapeutic) alliance, that connects (sometimes also separates) the patient, the therapist and the object, emerge out of these creative material entanglements of clay therapy. Nonetheless, there is nothing ‘symbolic’ (in the abstract representational sense of the term) about them. For instance, when a patient engages with clay in a sculpting task, meaning is enacted through the embodied process of acting with the material. This meaningful engagement with clay can produce very complicated affective dynamics (positive and negative).

Within art therapy there has been a diversity of approaches ranging from psychoanalytic and phenomenological to feminist and behavioural–cognitive, and from developmental and systemic to ecological and multi-modal/semiotic (Rubin, 2001; Betensky, 1995, 2001; Bar-On, 2007; Hogan, 2015; Martin et al., 2018; Brinck & Reddy, 2020; Van Lith, 2016). Looking at the available studies and clinical reports five therapeutic factors of clay-work are usually highlighted focusing on the ability of clay to facilitate (1) the expression of emotions, (2) catharsis, (3) deep expressions revealing unconscious material, (4) verbal and non-verbal communication, and last (5) objectification and symbolisation (Sholt & Gavron, 2006). As with other forms of art therapy emphasis can be placed both on the process (on how a piece is created) and the product (on what is created). In the former case, it is the handling and engagement with clay that primarily constitutes the therapeutic process. In the latter case, participants may be encouraged to create and then to discuss the issues expressed in their piece. However, what needs to be underlined, is that in both approaches (process oriented and product oriented) the major underlying assumption is the same: clay’s therapeutic impact is seen in the way it helps to express and reveal information about the patient’s mental world. Whether we translate the main problem and its possible causes in physiological (e.g., hormonal or neuronal) or psychological and behavioural processes, the fact remains that we are dealing with a *mental* problem caused by factors located *in* the patient. It is precisely this old dualistic division and topology of selfhood that is radically changing with the material engagement approach we propose.

## Discussion: Material Engagement and Embodied Therapeutic Practice in Psychiatry

Renewed scientific discourse can be noted regarding health-effects of environmental stressors (e.g. Reuben et al., 2022), referred to as “emerging research field of clinical ecopsychology” (Thoma et al., 2021) and the so-called ‘materialities of care’ (Buse et al., 2018).

Re-*thinging* psychiatry we set out to answer questions such as: are there any observable links between different kinds of material engagement and types of self-knowledge (bodily or narrative)? How does the experience of transparency, attachment, ownership, and connectivity become affected by changing the material ecology or the degree of immersion in material practices? Do they vary cross-culturally or according to gender?

We need to be pragmatic, inclusive and attentive in our approach, but also avoid reductionist methodologies (neuroscientific or other) and oversimplification at all costs.

One example is to look specifically at self-narratives evoked and constructed through shared discussions of artefacts and objects through art therapy and body-oriented psychological therapy interventions. With the help of things and materials the person could think the unthinkable or speak about the unspeakable. The operationalization of enactive signification (Malafouris, 2020; Malafouris et al., 2023; Koukouti & Malafouris, 2020) and creative *thinging* (Malafouris, 2014), processes which lie at the heart of art-therapy (although often misinterpreted and misunderstood), constitute a particularly appropriate psychotherapeutic approach to psychosis offering  a simple way to construct meaningful self-experiences and new forms of participatory sense making. Another example is the well-established utility of “therapeutic objects” in body-oriented psychological therapies. This involves a variety of objects and materials (e.g. stone, cloth, feather, etc.) for different (personalised) therapeutic aims, including fostering physical contact among group therapy participants or between therapist and client, enhancing body awareness through object-mediated touch, functional exemplification or playful exploration, and also utilising the associative affordance presented by the object’s characteristics. Exploring the surface and structure as well as functional utility of objects and tools invokes embodied cognitive processes, integrating sensory perception, affect, emotional responsiveness, movement and conceptual thinking. This opens ways to address fundamental disturbances of the basic self through the use of things with corresponding characteristics. Of course, from the perspective of MET, the objective and significance of these object-oriented interventions goes beyond the symbolic representation that characterize similar psychoanalytically informed relational intervention strategies. From a material engagement-perspective the main objective and focus of object manipulation is not on accessing supressed (affective) mental states that reside within individual humans (e.g. Vogt, 2006). Instead, the focus is on the enactive and affective co-constitution of new meanings between humans as well as between humans and things. In other words, material engagement interventions and practices can help us shape the conditions of possibility for meaning-making (e.g. Yatczak, 2019; Manzi et al., 2020).

Communication is one of the major challenges for psychiatric care in general, and participatory interactions using material engagement interventions may offer alternative non-verbal avenues of expression and non-verbal communication. Material belongings act as powerful media of enactive signification and thus can be as informative as self-testimonies and traditional forms of medical recording and documentation. Personal encounters with those objects, when attentively orchestrated by the therapist and the patient (or the patient’s family), can help re-enact (implicit) memories that could trigger therapeutic processes and enhance self-identity. It is also prompting the therapist to ask new questions and/or receive unexpected answers.

It is important to be clear here. Our claim is not that embodied (body-oriented and experiential) psychotherapeutic approaches based on creativity, body awareness and movement techniques, should be replaced with new ones based on object awareness and material engagement. Rather, what we suggest is that we should harness and incorporate the affective power of materiality for one treatment or another, and when successful, bring into being the necessary changes to facilitate their mobilization, and alliance, within established therapeutic settings. One such example of an integrated seamless approach is the Body Image Sculpturing (BIS) intervention and associate test (Aßmann et al., 2010; von Arnim, 2013, 2022). Joraschky and von Arnim (2009; Röhricht et al., 2014) outline the procedures of body image sculpturing to access multidimensional aspects of patient’s Body Image. Participants are provided with clay and asked to form a human figure with eyes closed thus facilitating tactile as well as proprioceptive experiences. They emphasise that the sculpture provides a highly ambiguous stimulus, thus opening a wide field for the participants’ engagement with material including subsequent therapeutic exploration of body-self states. Employing psychodynamic principles, the corresponding projective BIS-test uses a mixed-method approach to assess three core dimensions of the sculpture: “Proportionality, Completeness and Connectedness” in addition to the qualitative analysis of the body image directly after the plasticised figure is completed. Patients can be asked to “produce” a neutral figure or an image of themselves as an integral of their body experiences, using different materials (paper drawing, clay sculpture, their own bodies), followed by subsequent exploration of meaning-making (narratives) in relation to different modes of material engagement. In addition, the introduction of specific objects into individual integrative or psychodynamic psychological therapy could be evaluated. Alternatively, the comparison could be restricted to the intake (assessment) interview only, focusing upon establishing a clinical formulation of client’s difficulties.

What can we hope to achieve? It is important to recognise that every person's experience of mental illness is different. People have their own unique life histories, biographies and skills, leading to very personalised definitions of what constitutes treatment and recovery. They continue to make meaning of their lives through their attachments to and interactions with specific places, objects and people. People do not engage with the material world in general but with specific things, tools and aspects of the material environment that show up as meaningful or relevant to a specific context of activity (situatedness). Psychiatric disorders change the landscape of affordances (Rietveld & Kiverstein, 2014) in the way that ordinary habits and skills do. Material skilled practices facilitate attentive engagement, attunement and development of expertise (Malafouris & Koukouti, 2022), whereas psychiatric disorders typically hinder one’s relation to the world (often in a manner that involves a great deal of negative struggle and personal suffering). These are not absolute distinctions but context and culture dependent. Our modes of material engagement are relational and cannot be predicated on the basis of essentialist characteristics or classifications. There is no simple way to differentiate a depressogenic material ecology from one that in general facilitates positive emotional responses. However, understanding and comparing material environments may lead to the discovery of specific affordances that facilitate specific modes of engagement rather than others (for instance, creative vs. non creative forms of interaction). Clay, for instance, is a material that is usually associated with positive affect. But this does not exclude the possibility that this, generally perceived warm and playful, material cannot evoke responses or feelings of disgust, anxiety, or bodily discomfort and inconvenience. What is desirable for one person can be undesirable for another. However, as with other materials, clay has a certain creative and affective potential that, with guidance, can provide a valuable therapeutic anchor point for interventions. The argument we want to make is not that we can classify the material world into ready-made affective categories that somehow will exert similar effects and emotions to all bodies that engage with them. Rather, the argument we want to advance is that material matters and has an impact on human mental health and disorder that needs to be taken into serious consideration. That applies to the totality of the therapeutic environment, which includes the materiality of the place, in the sense of ‘lived space’ (Fuchs, 2007), its actions, relations and objects. This would lead us to considerations relevant to the possible role of ‘affect’ and ‘atmospheres’ as well as help to enhance or challenge conventional narrative approaches (Harries, 2020). Touching clay and touching a screen are two ‘external’ processes that could be equal parts of human ways of thinking and feeling. However, they share very little in common in terms of actual experience, affect and affordance.

Mundane material things, like a blind's person stick (Malafouris, 2008b), often far more efficiently than contemporary digital gadgets, can help people to re-orient and re-organise their cognitive landscape (neural and extra-neural) in a variety of ways. Introducing or creating scaffolds and material anchors (Hutchins, 2005) we could help actualise action affordances (Gallagher, 2018; Rietveld & Kiverstein, 2014) able to effect a more efficient utilisation of brain networks or stimulate the person's ability to recruit alternate brain networks that could enhance ‘cognitive reserve’ (Stern, 2002).

In summary: Things play an active role in enhancing self-specification, by anchoring and grounding self in action, strengthening agency against the weakening and temporal fragmentation of self-experience. Re-assembling the fragmented ‘intentional arc’^2^ and re-embodying self-experience by gathering space and time by means of *thinging* can also prove beneficial against the appearance of self-disturbances (such as thought withdrawal or insertion) as well as hallucinations and delusions. Things direct and situate bodily experiences in the environment. They are pivotal for embodied cognition, constituting and altering dynamically how individuals regulate their boundaries and how they relate to self and others. MET, drawing elements from old and new enactive–ecological trends in embodied cognitive science, provides a cross-disciplinary framework that allows re-*thinging* psychiatry by taking seriously the continuity and co-constitution of mind and matter.

## Footnotes

1 For a revealing autoethnographic account that challenges the discourses of loss and arguing for a more nuanced personal understanding of sense of self continuity within the lived experience of dementia see (Bryden, 2020).

2 This is the phrase that Merleau-Ponty uses to describe the underlying temporal continuity that permits the experiential continuity via ‘passive synthesis’, of the present with the immediate past (retention) and immediate future (protention). Maurice Merleau-Ponty, Phenomenology of perception (London: Routledge, 1962).
